# Enhancing data practices for Whole Health: Strategies for a transformative future

**DOI:** 10.1002/lrh2.10426

**Published:** 2024-05-30

**Authors:** Lei Guo, Kavitha P. Reddy, Theresa Van Iseghem, Whitney N. Pierce

**Affiliations:** ^1^ Whole Health VA St. Louis Health Care System St. Louis Missouri USA; ^2^ School of Interdisciplinary Health Professions Northern Illinois University DeKalb Illinois USA; ^3^ Department of Veterans Affairs VHA Office of Patient‐Centered Care and Cultural Transformation Washington D.C. USA; ^4^ School of Medicine Washington University in St. Louis St. Louis Missouri USA; ^5^ School of Medicine Saint Louis University St. Louis Missouri USA

**Keywords:** data analytics, patient‐centered care, read‐world data, whole health system

## Abstract

We explored the challenges and solutions for managing data within the Whole Health System (WHS), which operates as a Learning Health System and a patient‐centered healthcare approach that combines conventional and complementary approaches. Addressing these challenges is critical for enhancing patient care and improving outcomes within WHS. The proposed solutions include prioritizing interoperability for seamless data exchange, incorporating patient‐centered comparative clinical effectiveness research and real‐world data to personalize treatment plans and validate integrative approaches, and leveraging advanced data analytics tools to incorporate patient‐reported outcomes, objective metrics, robust data platforms. Implementing these measures will enable WHS to fulfill its mission as a holistic and patient‐centered healthcare model, promoting greater collaboration among providers, boosting the well‐being of patients and providers, and improving patient outcomes.

## 1 WHOLE HEALTH AT VA—A LEARNING HEALTH SYSTEM EXAMPLE

Whole Health is an approach to care that empowers and equips people to meet their health and well‐being goals and live life to the fullest. This approach combines conventional and complementary approaches to care for the whole person through multimodal interventions. It emphasizes coordinated care among patients, providers, instructors, peers, and institutions, with growing interest and research in its potential benefits for various health situations, including pain management, preventive health, and well‐being programs. Veterans Health Administration (VHA) can be considered and function as a Learning Health System (LHS) that systematically integrates external evidence with internal data and experience.^1^ This is evident by the establishment of the Whole Health System (WHS), the VA's approach to offering personalized, proactive, Veteran‐driven care.^2^ WHS matches the key components of the LHS as defined by the Agency for Healthcare Research and Quality (AHRQ), such as promoting the inclusion of patients, capturing data to improve care, and continually assessing outcomes to create learning and improvement feedback.^2^

### 1.1 Integral components of the Whole Health model

Whole Health is a resource for everyday life to enable people and communities to achieve their life aspirations and cope with change. Since 2010, VHA has been committed to delivering patient‐centered care, standing up the National Office of Patient‐Centered Care and Cultural Transformation (OPCC & CT). The initial efforts focused on innovative solutions for healthcare transformation that would deliver personalized, proactive, patient‐driven care. From 2010 to 2015, OPCC & CT learned valuable lessons regarding implementation efforts, feasibility, and essential components for delivering care, informing the development of the WHS of care. In response to the Comprehensive Addiction and Recovery Act in 2016's call for VHA to expand delivery of complementary and integrative health (CIH) approaches for Veterans with chronic pain and opioid addiction, VHA launched a pilot implementation of the WHS of care to 18 VA Medical Centers throughout the nation. This model aims to deliver CIH approaches in a patient‐centered manner, while also delivering the much‐needed well‐being and peer‐driven approaches. It allowed not only for more widespread Whole Health adoption, but the ability to evaluate implementation efforts as well.^2^ Since this initial launch in 2016, the WHS of care has expanded across all VHA medical centers with over 1.8 million Veterans accessing Whole Health approaches in Fiscal Year 2023. Building upon prior efforts, the VHA issued a formal directive in October 2023, outlining responsibilities and requirements for nationwide implementation of the WHS across VA Medical Centers. This directive further establishes feedback mechanisms and data‐driven evaluation metrics, aiming to promote continuous improvement and iterative refinement of the WHS model.

**FIGURE 1:**
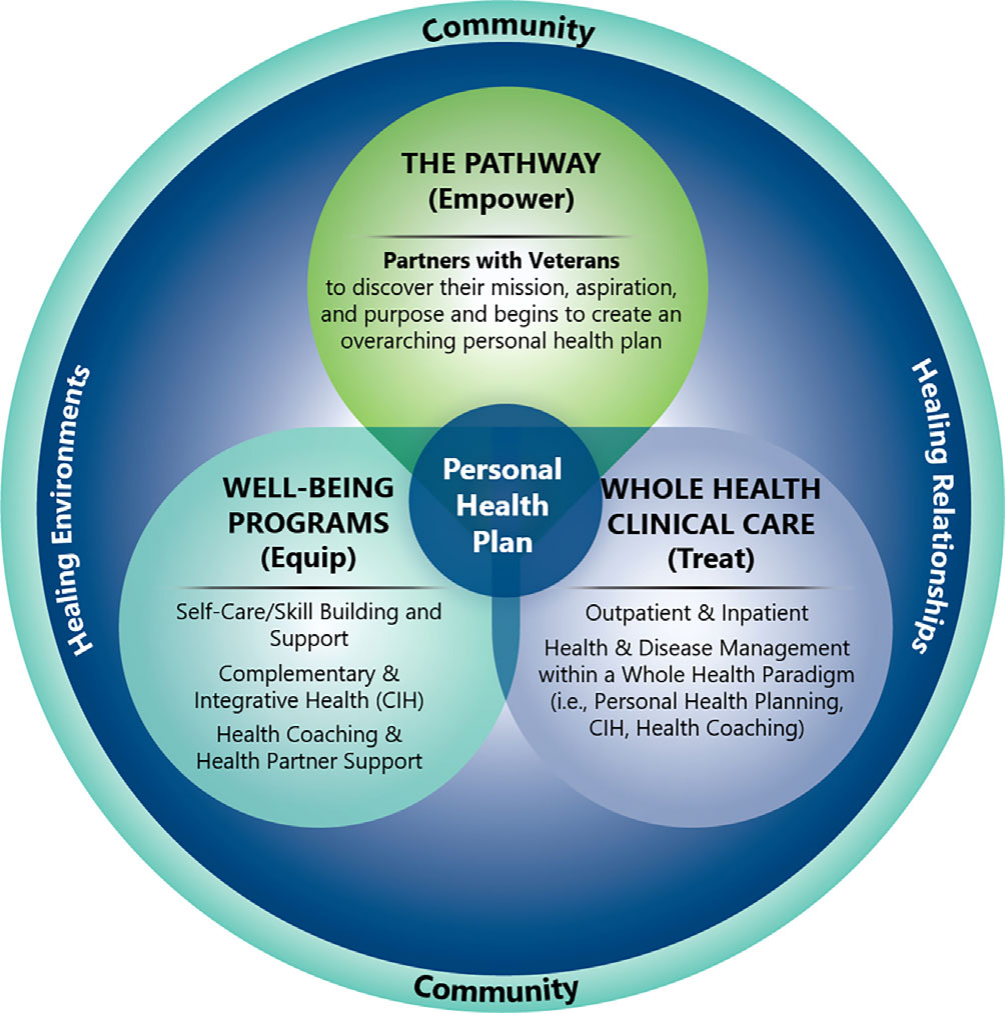
Key elements of VHA WHS.

There are three key elements of VHA WHS, as shown in Figure 1.

The Pathway serves as the foundational stage in Veterans' Whole Health journeys, where they are introduced to the concept of Whole Health and encouraged to reflect on their Mission, Aspiration, and Purpose (MAP). This phase enables them to explore diverse self‐care options and engage with Peer Partners or Whole Health Coaches to support their well‐being.

Well‐Being Programs are integral to the Whole Health System, offering Veterans the opportunity to acquire new self‐care skills through a range of activities. This may include mindfulness or nutrition classes, engagement with chaplaincy, and the exploration of Complementary and Integrative Health (CIH) approaches.

In Whole Health Clinical Care, a transdisciplinary team of clinicians, including providers, nurses, social workers, psychologists, pharmacists, and physical therapists, incorporates the Whole Health Approach into patient encounters. This holistic care model supports Veterans in creating personalized health plans, ensuring a patient‐centered focus on comprehensive well‐being and optimal health in all aspects of their lives, not just the absence of disease.

Beyond the now‐established VHA WHS, a universal definition from the National Academies of Science, Engineering and Medicine (NASEM) defines what Whole Health is—“physical, behavioral, spiritual, and socioeconomic well‐being as defined by individuals, families, and communities.”^3^ In other words, WHS is a holistic healthcare model that allows clinicians and instructors to practice complementary and integrative health, integrative health, lifestyle medicine,^4^ functional medicine,^5^ precision medicine,^6^ and so forth. In a multifaceted care model like this, there are challenges and opportunities related to data management, analysis, and utilization, all aimed at serving and enhancing the model's effectiveness.

## 2 DATA AND WHOLE HEALTH

Data, the key component in an LHS, connects evidence creation and collection through scientific research with clinical care that applies the most promising evidence to improve outcomes, echoing the classic three pillars of healthcare and LHS: clinical care, health learning/medical education, and research. The Office of Disease Prevention and Health Promotion of the U.S. Department of Health and Human Services has developed the Healthy People 2030 objectives to set data‐driven national goals to improve health and well‐being over the next decade.^7^ Additionally, measuring care progress and outcomes will be central to the learning and adaptive process for healthcare organizations and policymakers to scale and spread Whole Health.^3^

Furthermore, to help people, families, and communities achieve Whole Health, health informatics and data infrastructures shall support the five foundational elements of Whole Health: being people‐centered, comprehensive, holistic, upstream‐focused, equitable, accountable, and grounded in team well‐being.^3^ The NAM report highlighted several existing gaps and roadblocks. First, there is a lack of standardized metrics to evaluate the achievement of Whole Health and Whole Health care. Second, challenges in studying care delivery can impede data collection and analysis. Finally, when designing a Whole Health model, the healthcare system may need to balance patient outcomes and operational costs with sustainability as a critical consideration.^3^ These burdens, along with variations in data collection practices and limited interoperability among healthcare systems, may contribute to the data issues.

Real‐world evidence (RWE) is defined as “information on health care that is derived from multiple sources outside typical clinical research settings.”^8^ While the clinical trial has traditionally been the gold standard for delivering science‐based and medically validated care, it fails to integrate practical outcome data (e.g., Real‐World Data, RWD), such as data collected from other WHS providers delivering concurrent care or patient‐reported outcomes (PRO) that, collectively, create RWE and supports WHS providers in the development of treatment planning and inform researchers to monitor and track patient outcomes.

Clinical practice often generates large amounts of data at the patient level. Over the years, national and local level initiatives have shifted toward collaborative treatment planning with the veteran, the use of shared decision‐making between patient and provider, and the use of metrics to support efficacious treatment delivery (Table 2). As such, various approaches (e.g., such as the Assessing Circumstances & Offering Resources for Needs [ACORN] and the cloud‐based virtual platform Behavioral Health Laboratory [BHL])^9^, ^10^ were developed to streamline data processing, capture veteran perspective, and allocate resources to veterans in a timely manner. Additionally, health factors are now captured during patient encounters, which help quickly identify the specific WH interventions the veteran utilizes during their appointments. Biometric measures, such as weight, blood pressure, lipid panel, and HgA1c, capture the big picture of health markers relevant to assessing the whole person and, when used collaboratively, can help to inform a more comprehensive approach to long‐term care plans. Evidence‐based care is routinely used within behavioral health treatment plans; data captured through veteran reports can be an important aspect of whole‐person assessment, but only if all veteran providers easily access the data. From a Whole Health perspective, health outcome data, including behavioral health measures, can help to identify situational factors relevant to symptom exacerbation and potentially help to prioritize treatment goals that more accurately align with the veteran's overall health and well‐being.

Beyond regular clinical outcome data, WH also focuses on the patient's level of engagement and activation, aiming for improvements in their quality of life. Engagement refers to how often a Veteran utilizes WH resources and regularly communicates with providers. Activation refers to how actively the Veteran participates in healthcare management, which is an important indicator of the success and effectiveness of patient‐centered care. For example, at the STL VA Whole Health, health coaches utilized the Patient Activation Measure (PAM‐13) to assess a Veteran's knowledge, confidence, and skills in managing their health.^11^ This helped to tailor care plans by considering the patient's readiness for specific CIH approaches and other healthcare resources, including Whole Health programs. Understanding patient activation levels allows WH providers to communicate effectively with patients, explaining options and potential benefits and drawbacks of various approaches in a way that aligns with the patient's level of understanding and engagement. Moreover, the STL Whole Health team also uses the Well‐Being Signs, a 3‐question quantitative instrument designed to assess veterans' daily functioning.^12^ It serves a dual purpose to inform treatment planning and referral decisions, including those for Whole Health programs.

Leveraging health data collected across various VA care teams aligns with the principles of the LHS and complements WH practices. One example is the patient care assessment system (PCAS). It is a web‐based application that helps identify, manage, and coordinate patient care. PCAS integrates key data from multiple sources and summarizes patient risk factors. It can create suggested care plans for managing certain comorbidities like liver and cardiometabolic, and so forth. WH data points can be integrated into this platform to provide personalized, patient‐driven care and support the provider's decision‐making. These efforts represent the ever‐evolving nature of the WHS as a learning health system, constantly seeking to improve patient care through data collection, analysis, and adaptation.

## 3 COMMON RWD CHALLENGES AND BURDENS OF WHOLE HEALTH

### 3.1 Data capacity

Data capacity refers to the availability and suitability of data, as well as the maximum amount of data that a storage system, database, or technology infrastructure can effectively handle, store, or process within a specified timeframe or resource constraint.^13^ While technical limitations define the raw data storage ceiling, WHS, as an LHS, thrives on robust data capacity, allowing comprehensive integration of medical, social, and patient‐reported data, fueling personalized care plans and proactive interventions that address the whole person. Research indicates that the utilization of patient‐reported measurements can assist clinicians in selecting care plans grounded in the best evidence, such as integrative oncology.^14^, ^15^ However, the inconsistent and inaccurate data capture by different interdisciplinary clinicians across clinical data, social determinants, and patient‐reported outcomes, a common challenge in many healthcare systems^16^, ^17^—can be a fundamental data capacity bottleneck that limits the ability to address patients' whole person needs. Furthermore, incomplete and variable categorization of data on health factors and social determinants of health can hinder care outcomes, especially when many practices have now transitioned to digital health, such as telehealth, mobile health, and wearable technologies.^18^ On the other hand, mistakes during data collection, such as recording incorrect values or missing data points, can introduce bias, impact the care outcomes, and even delay the treatment.^19^, ^20^ Limited physical storage capacity and inconsistent data availability for WHS clinicians present considerable barriers to the integration of Whole Health initiatives within traditional clinical practice.

### 3.2 Data bias

Data bias refers to systematic and prejudiced errors in data collection, analysis, or interpretation that result in skewed or inaccurate outcomes, often leading to unfair or unrepresentative conclusions. WHS hinges on PRO measures empowering patients to reflect on their physical, emotional, and spiritual well‐being to individuals to make informed treatment choices and communicate their preferences. These outcomes aid in assessing treatment effectiveness, enhancing quality of life, and driving research, while ensuring integrative health aligns with patient needs and values at a systems level. In fact, they contribute tremendously to healthcare system transformation and enhancing the value proposition for investing in the WHS.^21^ On the other hand, individual‐level PRO data can be subject to various biases, including recall bias (inaccurate recollection of information) and response bias (influenced patient responses).^22^, ^23^ Recall bias may lead patients to inaccurately remember and report their previous treatments or therapies, potentially omitting essential information. Such omissions can hinder healthcare providers' ability to develop comprehensive care plans tailored to patients' needs. Furthermore, response bias can influence how patients describe their experiences with integrative treatments, potentially leading to an overly positive or socially desirable portrayal.^24^ Mitigating these biases is crucial to ensuring patient care remains truly holistic, evidence‐based, and focused on optimizing health well‐being. It is vital to implement standardized assessment tools, provide cultural sensitivity, and offer diverse data collection methods while ensuring healthcare providers receive training in unbiased data interpretation. Additionally, inclusive accessibility^25^ and patient engagement^26^ coupled with a LHS feedback loop,^27^, ^28^ can help mitigate biases and improve the overall quality and relevance of PRO data.

### 3.3 Data integration and standardization

Data Integration is combining data from various sources to create a unified dataset. At the same time, data standardization involves establishing uniform formats, definitions, and structures for consistent and compatible data handling and exchange. This is often challenging because of the variation in the training and certification requirements for providers delivering a wide range of services in WHS. The absence of clear guidance for outcome measure selection is insufficient consideration of the clinical significance of PRO results.^19^, ^29^ Additionally, combining data from various sources, such as electronic health records, surveys, and wearable devices, can be complex,^29^ and can hinder the ability to gain a comprehensive view of a patient's health. Furthermore, the lack of standardized data formats and terminologies between healthcare systems can create continuity of care challenges and complicate consistent and effective assessment and evidence‐based treatment planning practices when patients seek care in a different system.^19^

### 3.4 Data volume and handling

Data volume and handling refer to the quantity and management of data, respectively, focusing on efficiently storing, processing, and analyzing large amounts of data. WHS practices generate substantial amounts of data due to a holistic approach, which considers not only medical records but also PRO, lifestyle factors, and complementary therapies.^30^ This complex data collection provides a rich source of information for healthcare analytics, enabling personalized treatment plans and data‐driven insights to improve patient outcomes.^31^ Still, it also poses a unique challenge within the context of WHS. This challenge becomes particularly pronounced as the field embraces the emerging trend of incorporating new technologies such as telehealth,^32^, ^33^ wearable health devices,^3^ and digital health,^34^ which are all integral to fueling AI‐driven insights. As practitioners navigate this data‐rich landscape, they face the challenge of unlocking the full potential of the abundance of collected data while upholding the core principles of WHS, where the patient's well‐being remains at the forefront of care.

### 3.5 Data security and privacy

Data security and privacy refer to measures and practices aimed at safeguarding sensitive data from unauthorized access, disclosure, alteration, or destruction while ensuring that individuals' personal information is protected and used appropriately. Protecting sensitive health information can be a significant concern.^35^ In WHS, data security and privacy assume paramount importance as the field navigates the integration of diverse data sources and innovative technologies to provide holistic and patient‐centered care. For example, the interconnectedness of electronic health records, wearable devices, and PRO necessitates rigorous data security measures to shield sensitive health information from breaches.^36^ The challenge lies in ensuring that while these WHS practices harness the potential of advanced technologies, patients' data remains safeguarded in compliance with privacy regulations.^37^ Moreover, respecting individuals' privacy rights and gaining their trust are essential to maintaining the integrity of WHS, where the holistic well‐being of the patient is not only the goal but also a foundational principle. WHS providers are tasked with striking a delicate balance, harnessing the benefits of data‐driven insights while upholding the patient‐centered approach that defines their practice, thus underscoring the critical importance of data security and privacy in this evolving healthcare landscape.

Table 1 provides examples of common data issues and burdens encountered in WHS clinical practice.

**TABLE 1:** Common data issues and burdens in Whole Health clinical practice.

Data issues	Example of related Whole Health clinical practices	Burdens to first‐line providers and impact on outcomes
Data capacity	*Acupuncture*: Collecting and analyzing data on acupuncture treatments, including different types of PROs and objective measures of pain or other symptoms. *Issue*: Challenge to apply the real‐time analyzed PRO to assist clinical decisions at the point‐of‐care.	Limited point‐of‐care access to real‐time data hampers whole‐person and holistic assessment. Lagging data delays shared decision‐making, limiting patient engagement. Information silos due to limited real‐time data stalls interdisciplinary interventions, impacting treatment effectiveness.
Data bias	*Mindfulness*: Assessing the effectiveness of mindfulness interventions using PROs and other measures of mindfulness, such as self‐reported awareness and acceptance that may have biased data elements. *Issues*: Failure to collect other variables that may contribute to outcomes	
Data integration and standardization	*Weight management and functional nutritional counseling*: Combining data from multiple sources such as digital food diaries and physical activity tracking and the electronic health record (EHR) notes of an interdisciplinary team that may include dietitians, health coaches, integrative medicine physicians, and behavioral health therapists to create a comprehensive picture of a patient's nutritional status and factors influencing dietary habits. *Issues*: Difficulties in data integration from Portable or wearable equipment.	
Data volume and handling	*Tele‐Whole Health*: Collecting and analyzing data from Tele‐Whole Health visits, including PROs, vital signs, and clinical assessments. Issue: Limited data processing and analysis in real‐time.	
Data security and privacy	*Peer Partner*: Safeguarding information from collected personal histories veterans share and protecting sensitive personal data from unauthorized access, disclosure, or alteration while ensuring that patients' privacy rights are protected. *Issues*: Lack of access to a HIPAA compliant cloud space for veterans to access meditation or mindfulness recordings.	

## 4 UNDERSTANDING DATA CHALLENGES IN THE CONTEXT OF WHOLE HEALTH'S CORE PRINCIPLES

**FIGURE 2:**
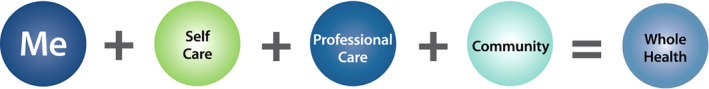
Whole Health equation.

WHS recognizes a person's uniqueness, placing them at the core of their care. This patient‐centered approach empowers individuals through mindful awareness and self‐care, with the support and guidance of a multidisciplinary team of professionals who may combine conventional and complementary approaches. Additionally, the community plays an integral role in embracing and reinforcing this holistic approach to healthcare (Figure 2). Data challenges within WHS may manifest across different core elements of this approach. These issues become evident as clinicians strive to align data processes with the fundamental principles that define WHS. Understanding how data intricacies impact various facets of healthcare delivery is crucial in addressing these challenges and optimizing the quality of care provided within the WHS context. These data‐related hurdles are not confined to a single element but can extend across multiple aspects of WHS, reflecting the complex nature of this approach. From a broader perspective, these insights into data challenges can also inform and benefit the LHS, emphasizing the interconnectedness of data optimization efforts across healthcare domains.

### 4.1 Patient‐centered

Patient compliance and self‐reported data pose challenges and opportunities within the patient‐centered WHS approach. While WHS prioritizes patient‐centered care, encompassing the broader concept of people‐centered care as it expands to cater to diverse health populations, patient activation and compliance levels can notably impact treatment and health outcomes.^38^, ^39^, ^40^ This impact sometimes arises from the limited availability of reliable data to clinicians, hindering the ability to provide tailored care effectively. Thus, maintaining a balanced and robust data and information infrastructure is vital to ensuring the quality of care within WHS. Additionally, many common pathways and well‐being practices, including Complementary and Integrative Health (CIH) interventions, are closely associated with behavioral interventions that rely on patient‐reported outcomes (PRO).^41^, ^42^ However, PRO in these contexts is often deemed unreliable due to issues such as recall bias or social desirability bias, as demonstrated in various studies.^43^ Consequently, the reliability of self‐report data can pose challenges in measuring and assessing outcomes within the holistic care model promoted by WHS.

Variations in regional cultures and social deprivation represent another dimension of data challenges within the patient‐centered context of WHS. Cultural and social factors exert significant influence over the collection and analysis of data, thereby impacting the delivery of holistic care and achieving equitable health outcomes. Limitations of the analysis tool for the PRO, including underlying bias due to limited data and the inability to capture veterans' specific needs and experiences, can further complicate efforts to address the potential challenges disadvantaged veteran populations face. These variations underscore the need for sensitivity to cultural diversity and the unique social contexts in which patients are situated.^44^ Despite the limitations, PROs still shall play fundamental roles in WH care as providers can obtain valuable insights about Veteran's self‐efficacy, progress of self‐care and health management, healthcare resource utilization, and so forth. Understanding these nuances in data collection and analysis is essential for ensuring that WHS practices remain responsive to individuals' specific needs and circumstances within diverse populations, aligning with the patient‐centered ethos of WHS and similar models.

### 4.2 Comprehensive and holistic

WHS's comprehensive and holistic element introduces a unique set of challenges concerning data management and utilization. One of these challenges is related to data integration, which involves amalgamating data from diverse sources to gain a comprehensive view of a patient's health and well‐being. WHS emphasizes the importance of considering various aspects of a patient's life, from clinical health to social determinants, making integrating such disparate data sources essential. However, this integration process can be intricate and time‐consuming, requiring effective strategies to streamline data flow and maintain data integrity. Discrepancies in the collection of quality‐of‐life data and satisfaction with SMART (specific, measurable, action‐oriented, realistic, and timed) goals can frequently constrain the intervention options available to WHS providers.^19^, ^28^

Another significant issue is the lack of standardized metrics for holistic outcome assessment.^28^ In the holistic care model promoted by WHS, patients have the flexibility to focus on addressing immediate health concerns, chronic conditions, and social needs simultaneously. While this patient‐centric approach enhances care delivery, it introduces data challenges related to outcome measurement. Furthermore, while objective metrics effectively capture many aspects of health, certain health behavior changes, such as perceptions of self‐care efforts, prove more challenging to quantify. For some patients, self‐efficacy improvements may be transient, potentially leading to discontinuing their Whole Health journal unless providers offer timely encouragement and support. Given that such qualitative data is solely patient‐reported, PROs are essential for effective care planning and delivery. However, the absence of standardized objective metrics hinders the consistent and effective assessment of holistic outcomes across different patient cases. Developing such metrics is essential to effectively gauge the impact of WHS practices and ensure that patients receive comprehensive care tailored to their unique needs.

Additionally, WHS's comprehensive and holistic nature emphasizes the importance of long‐term data collection and analysis to support holistic wellness. Patients may choose to address acute and chronic conditions over extended periods, making long‐term data essential for tracking progress and optimizing care. However, collecting and analyzing long‐term data in WHS can be challenging,^28^, ^45^ requiring sustainable data infrastructures and practices to ensure data accuracy, security, and accessibility. These data challenges align with emerging issues in data management, including data security and privacy, data volume and handling, data integration and standardization, data bias, and data capacity. Addressing these rising issues is crucial for enhancing the comprehensive and holistic care provided within the WHS framework while maintaining patient privacy and data accuracy.

### 4.3 Upstream‐focused

The upstream‐focused element of Whole Health highlights the importance of preventive measures and addressing health determinants at their source at the patient level by offering guidance and support, such as community‐based programs aimed at addressing SDOH like social isolation and food insecurity. This approach necessitates a robust data infrastructure to support comprehensive data collection and analysis. However, a notable data challenge in this context is the presence of data resource constraints. For example, Veterans' community program participation activity data may heavily rely on PRO measures, which are often not exchanged between the programs and the WHS system automatically. Thus, limited resources can significantly impact the ability to collect comprehensive data, especially in the context of data modeling. Data resource constraints can manifest in various ways, including limited access to essential data sources,^46^ lack of communication with patients and other stakeholders regarding the measurements,^47^ insufficient funding for data collection initiatives,^48^ and inadequate technology infrastructure.^49^ These constraints hinder the comprehensive data gathering needed to understand and address the upstream factors influencing health outcomes (Table 2).

**TABLE 2:** Clinical data collection and utilization examples in Whole Health.

Category	Data collection level	Measurement examples	Application in shared decision‐making and Whole Health care
Assessing circumstances & offering resources for needs (ACRON)	National	Social determinants of health (SDOH)	Systematically screen Veterans for social needs in nine social risk domains; Provide clinical teams with real‐time information about veterans' unmet needs. Address identified needs through the provision of resources and referrals.
Behavioral health laboratory (BHL)	National and local	Patient Health Questionnaire‐9 (PHQ9), Patient‐Reported Outcomes Measurement Information System (PROMIS), depression, domestic abuse, and suicidality screens	Collecting mental health assessment data captured during structured interviews with patients provides customizable patient tracking, health screening, decision support, and assessment tools and generates patient and program‐level data to inform providers and patients.
Health factors	National and local	CIH approaches, coaching, peer‐facilitated classes	These give a quick snapshot into what elements of the Whole Health system the Veteran is engaging in versus a time‐consuming chart review of all notes over the past year.
Biometric measures	VA STL Whole Health (local)	Weight, lipid panel (including HDL cholesterol, LDL cholesterol, and triglycerides), and HbA1c (glycated hemoglobin).	Evaluating these measures provides a comprehensive assessment of cardiometabolic risk factors for informed preventive healthcare strategies and potential long‐term care plans.
Pain measures	National and local	Pain measures as part of the VA Cognitive Behavioral for Chronic Pain (CBT‐CP) protocol. For example, Concerns About Pain (CAP) Scale, West Haven‐Yale Multidimensional Pain Inventory—Interference Scale (MPI‐INT), and Pain Numerical Rating Scale (NRS).	CAP measures an individual's level of pain catastrophizing and is often considered a consistent predictor of disability. MPI‐INT measures Veteran's perception of how much pain is interfering in multiple life domains (socialization, work, daily activities, and relationships with others). NRS assesses the pain level, rating from average, worst, best pain over the past week.
Suicide measures	National and local	Columbia – Suicide Severity Rating Scale (C‐SSRS); Comprehensive Suicide Risk Evaluation (CSRE)	Allow providers and patients to identify potential suicidal risk factors.
Evidence‐based engagement approaches	VA STL whole health (local) and national	Patient activation level and well‐being signs.	These measures facilitate the tailoring of care plans to match their receptiveness to specific CIH approaches and other healthcare resources, including Whole Health programs.
The patient care assessment system	National and local	Patient visits reminder; suggested care steps for managing certain comorbidities like liver and cardiometabolic	Integrated data platform for clinicians to see a snapshot of how their veteran is doing. Whole Health data points can be integrated into this platform.

Moreover, within the upstream‐focused approach, data challenges extend beyond resource constraints. One critical concern is the need for practical data interpretation and actionability.^50^, ^51^ While collecting data is essential, it is equally important to interpret it accurately and derive actionable insights that inform holistic treatment planning. This requires specialized skills and resources in data analysis and interpretation based on WHS principles. For instance, many WHS clinicians and providers lack specific training in data analytics tasks, particularly those involving the emerging field of RWE and its focus on personalized approaches. This lack of preparation can hinder their ability to transform raw data into actionable information that effectively informs preventive. Additionally, ensuring data quality and accuracy is vital to the success of upstream‐focused efforts, as data errors or biases can lead to misguided interventions.^52^ Therefore, addressing data issues related to resource constraints, interpretation, actionability, and data quality is essential to realizing the potential of the upstream‐focused element in a WHS and achieving meaningful improvements in health outcomes.

### 4.4 Equitable and accountable

The “Equitable and Accountable” component of WHS highlights the critical importance of tackling health disparities and ensuring healthcare services' just and equitable provision. Nevertheless, achieving equity in healthcare demands a meticulous examination of data‐related concerns, particularly bias and differences. The existence of data bias and health disparities pose substantial challenges within this domain.^53^ It is imperative to rectify biases in data collection to guarantee that healthcare decisions are equitable and grounded in precise information. These biases can stem from various sources, including unequal access to healthcare resources, cultural biases ingrained in data collection methods, or systemic imbalances in data representation.^54^ The correction of such biases is paramount in delivering impartial and equitable care to all individuals, irrespective of their background or circumstances.

Data privacy and security are of paramount importance within the equitable and accountable facet. Ensuring the confidentiality and security of patient data is not only a legal and ethical responsibility but also essential for upholding accountability in healthcare delivery.^55^, ^56^ Patients must have confidence that their sensitive health information remains safeguarded to promote transparency and accountability within the WHS. Cultivating this trust necessitates implementing robust data security measures encompassing encryption, stringent access controls, and adherence to data protection regulations.^57^, ^58^, ^59^ The pursuit of healthcare equity mandates that all patients, regardless of their background, can place trust in the confidentiality and security of their health data. Addressing these data‐related challenges within the equitable and accountable element is foundational to establishing a WHS that prioritizes fairness, accountability, and the elimination of health disparities.

### 4.5 Grounded in team well‐being

One of the primary data‐related issues in this context is collecting and managing data pertaining to healthcare providers' well‐being.^54^, ^59^ Providers often face high levels of stress and burnout in their demanding roles, which can harm their health and the quality of care they provide.^60^ Gathering data on provider well‐being is crucial for understanding and addressing these issues. However, it also raises concerns about data privacy and confidentiality. Ensuring that the collected data is anonymized and securely stored is essential to protect providers' privacy while still gaining valuable insights into the factors affecting their well‐being.^61^

Another significant data challenge lies in measuring the effectiveness of provider education programs in improving team well‐being.^62^ Data analytics can play a pivotal role in identifying gaps in education and training, enabling healthcare organizations to tailor programs to meet specific needs. However, data accuracy and relevance are critical considerations here.^63^ The data used to assess provider education should align with the real challenges and requirements of the healthcare environment.^29^ Inaccurate or outdated data can lead to ineffective educational initiatives, hampering efforts to enhance team well‐being. Therefore, maintaining data accuracy and relevance is paramount for supporting the continuous improvement of provider education and, by extension, strengthening team well‐being within the framework of Whole Health care.

## 5 PRACTICAL STEPS TO ADDRESS THE ABOVE ISSUES

We recommend that the WHS team take the following practical steps into consideration when addressing the data issues.

### 5.1 Facilitating interoperability

Interoperability, the ability of two or more systems to exchange health information and use the information once it is received,^64^ is paramount in both WHS and learning health systems. In a learning health system, interoperability enables the aggregation of diverse data sources, leading to more robust research and analysis of WHS interventions and reduce the data integration and standardization burden.^65^ It supports the goal of continuous improvement by facilitating the seamless exchange of health data across the healthcare ecosystem. To address these issues, the VA has published a strategic plan to create seamless and secure interoperability and health information exchanges of Veterans' health information among the VA, the Department of Defense, and the community (Office of Information and Technology, 2020). The strategic plan includes developing and implementing the Fast Health Interoperability Resources (FHIR) data architecture, using Application Programming Interfaces (APIs) and cloud technologies, and incorporating cybersecurity and privacy tools. For the local Whole Health sites, efforts should be made to enhance these goals to enable providers and veterans to deliver care plans with the best evidence.

### 5.2 Incorporating patient‐centered comparative clinical effectiveness research

WHS, with its emphasis on patient‐centered and evidence‐based practices, stands to benefit immensely from the incorporation of clinical‐effectiveness research (CER) and RWD.^66^ Comparative effectiveness research is a strategy that focuses on the practical comparison of two or more health interventions to discern what works best for which patients and populations. It enables a more tailored and responsive approach to patient care, where treatments can be personalized based on individual needs and preferences using patient‐centered health data, including conventional PRO, and wearable health devices, and telehealth data. By leveraging RWD, WHS practitioners gain insights into diverse patient experiences and outcomes, facilitating the optimization of treatment plans in real‐time.^18^, ^67^ This dynamic and adaptive approach, fueled by continuous monitoring, ensures that Whole Health clinicians can build clinical data capacity to deliver effective and safe integrative therapies addressing the multisectoral needs indicated. Furthermore, it fosters a culture of continuous improvement, where treatment protocols evolve based on the latest evidence, ultimately leading to better patient outcomes.

The integration of CER and RWD also offers the potential to validate integrative therapies and solidify their place in mainstream healthcare. By collecting and analyzing RWE, the field can demonstrate the effectiveness of WHS approaches, gaining recognition and acceptance within the broader medical community. Additionally, the longitudinal nature of RWD enables the assessment of long‐term impacts, unveiling trends and potential long‐term benefits that may not be immediately apparent through traditional clinical trials.^67^ This extended perspective contributes to a more comprehensive understanding of WHS interventions. Moreover, the collaborative aspect of this integration encourages interdisciplinary cooperation among healthcare providers, fostering a holistic approach to patient care and promoting the exploration of complementary and integrative practices across various medical specialties.

### 5.3 Advanced data analytics tools and data platform

Advanced data analytics tools, such as machine learning, deep learning, and artificial intelligence, can empower healthcare professionals to extract rich insights from complex datasets, track performance metrics, and monitor patient outcomes with unprecedented precision. They can impact the clinical care in WHS to reduce the data analysis cycle, improve the predictive modeling, optimize, and discover new interventions using RWD. Real‐time dashboards and reports further enhance decision‐making and quality improvement efforts.^68^ In WHS, the synergy of data analytics and AI can enable the assessment of various integrative therapies' effectiveness in real‐time, incorporating patients' insights while identifying nuanced success patterns for specific patient populations. Despite the previously stated limitations of PROs, WH data analytics should incorporate more objective measures, including biometrics, functional ability (e.g., daily living activities), healthcare utilization, medication usage, and so forth, to accommodate the patient's personal needs. This will also allow for evaluation of the effectiveness of WH interventions from a population perspective, further improving efficacy. Meanwhile, these multifaceted tools support ongoing research and quality improvement initiatives within a learning health system, enabling providers to adapt and optimize WHS practices based on sophisticated data‐driven insights.

Moreover, it is imperative to construct a data platform that considers interoperability, big data analytics, and emerging AI technologies. Given the sensitive and intricate nature of health data, there are only a few practical examples of platforms capable of merging and processing people‐centered big data.^69^, ^70^ Beyond the common features such as security and privacy, governance, access and permissions, and data quality assurance,^71^ the Whole Health specified platform shall consider the following elements:

#### 5.3.1 Interoperability and integration

Ensure the platform integrates data from various sources, including electronic health records (EHRs), wearable devices, and patient‐reported data. Data normalization and standardization are crucial to enable data exchange between different healthcare systems, improving care coordination and continuity.

#### 5.3.2 Visualization

It provides the tools to convert complex, multidimensional data into visualized tools that help providers from various WHS care fields process and utilize the clinical information. It can also help providers explain progress to veterans in an easy‐to‐understand format.

#### 5.3.3 Data analysis tool

It shall be equipped with cutting‐edge algorithms designed for WHS, enable real‐time data processing, and ensure data quality, thus fulfilling the essential requirements for effective Whole Health analytics.

#### 5.3.4 Data reporting

The tools can generate reports that facilitate better comprehension for various stakeholders, including interdisciplinary providers, patients, caregivers, health system administrators, and policymakers.

Ultimately, bridging data gaps and empowering WHS professionals are key to overcoming challenges like data bias and volume. Interoperability break down silos, allowing diverse data sources to be seamlessly integrated and analyzed, reducing bias and informing personalized interventions. Patient‐centered research further combats bias by incorporating individual needs, while advanced analytics tools handle the increasing data volume effectively. However, data security and capacity remain crucial concerns. Robust governance and scalable infrastructure are essential to ensure ethical data use and keep pace with evolving needs.

The core principles of the LHS are embodied in this continuous cycle of identifying challenges, developing solutions, and integrating them into practice. By acknowledging the limitations of current metrics for holistic care, Whole Health paves the way for innovation. Efforts outlined above, such as fostering interoperability and leveraging advanced analytics, demonstrate the data‐driven approach inherent to the LHS. This continuous learning loop ensures that Whole Health care delivery evolves to meet the ever‐changing needs of veterans. While challenges like data security and capacity remain, the collaborative spirit and focus on improvement within the LHS offer an innovative future for Whole Health. This data‐driven approach holds the potential to transform healthcare delivery for veterans, creating a future filled with personalized, effective care.

## 6 CONCLUSION

In conclusion, our exploration of major data issues within the context of the Whole Health System as a Learning Health System has shed light on the multifaceted challenges faced in achieving comprehensive healthcare. We dissected these challenges through the lens of the five foundational elements of Whole Health, allowing us to gain a deeper understanding of the intricate interplay between data and holistic well‐being. While Whole Health temporarily faces challenges in developing metrics tailored to personalized, holistic care models, the issues and efforts discussed here exemplify the innovative and data‐driven nature of the Learning Health System and Whole Health. Through our analysis, we have proposed a range of key solutions that emphasize the importance of data standardization, security, ethics, and accessibility, all aimed at fostering equitable and accountable healthcare.

## FUNDING INFORMATION

This work was not supported by grant funding.

## CONFLICT OF INTEREST STATEMENT

Kavitha Reddy and Theresa Van Iseghem are the principal investigators of an NIH clinical trial. All the other authors declare that they have no conflicts of interest.
